# APACHE and Brazilian intensive care units

**DOI:** 10.1590/S1516-31802003000200001

**Published:** 2003-03-05

Two major questions arise regarding disease scoring in intensive care units: the resources/benefit relationship per disease and patient prognosis. Discussion of the cost of treating patients is a very difficult matter. However, it is a very hot theme for managers: they need to do their best for people with a limited amount of money. At some point they need to decide on the best option for the healthcare system to invest in and get the best results for a community, city or country. Thus, the only reasonable way to make a decision is by analyzing prognosis scores so as to decide on the best cost/benefit.

The Brazilian public healthcare system presents a scenario in which the emergency rooms are not in a condition to treat all their patients’ demands and there are insufficient intensive care unit beds in hospitals. The question is whether more resources should be allocated to these areas. The answer is surely, yes. However, we need to be attentive to the fact that most of these patients could be prevented from having acute clinical decompensation and consequently requiring more difficult and expensive treatment. Where should such prevention be done? Within the primary care. Adequate treatment at the outset of a disease or during the initial period when the disease may be stabilized easily will avoid chronic complications for the patient. This is therefore the best time for treatment to be given. This conclusion thus gives a new direction for the resources: towards primary care.

If patients receive good care at the primary level, there will be a reduction in acute decompensation due to diseases, and lower patient demand for emergency rooms. In the case of outpatient treatment, even if the drugs and complete treatment are paid for by the public system it will be less expensive than treatment in a hospital. This does not mean that we do not need any more hospitals or intensive care units, but we can save money and lives if the resources are better invested within the healthcare system.

One possible reason why the clinical patients in the study reported in this issue^1^ had higher mortality than in the APACHE (Acute Physiology and Chronic Health Evaluation) score prediction may be that the patients in this study came from lower social strata. Thus, those patients had previous complications from chronic diseases (diabetes, systemic hypertension), which may have compromised future evolution. APACHE scores cannot take into account the level of damage to cerebral or coronary arteries or the microcirculation. Thus, patients who had not had continuous care would have chronic complications when they presented at a hospital, thereby determining that their evolution would be worse.

The prognostic prediction is the main feature of scoring systems. It helps physicians to know whether their actions are producing good results or not.^2,3^ However, there are some factors that may change the scores for a single patient, and for a specific intensive care unit population.

Firstly, it has been shown that there is some variability in scoring that depends on the physician taking the data.^4^ This can be minimized after training the whole team to acquire the data in an equal way. Therefore, the intensive care units always need to have the same person doing the data input into the databank, or they should train every member of the team to do this.

Secondly, the initial study that analyzed the physiological parameters and validated the APACHE II score did this for a wide range of diseases. Knaus et al. found correction factors for some diseases, but not for all situations.^5^ The kinds of diseases in the study might not be the same as in a new intensive care unit that wanted to use the score. Also, for some diseases like pneumonia, correlations between them and social strata have been shown.^6^ For people from a poor social situation there is worse evolution for the same disease. Also, it has been shown that there is a relationship between race and survival among patients in intensive care. In that study,^7^ black patients were almost three times more likely than white patients to die in hospital following admission to the intensive care unit.

These considerations should put us on alert regarding how to analyze differences between APACHE II predictions and recorded results. Also, such points could explain the differences found among the clinical patients in the study published in this journal.^1^

It could be argued that these differences signify some difference in technical or medical quality. However, it can be seen that the surgical group of patients in the study^1^ evolved in accordance with the APACHE II predictions. This result maybe allows the hypothesis of technical differences to be discarded.

All these questions have highlighted the need to customize or validate the APACHE II system for the service where it will be used, in order to obtain more precise analysis of the results from that intensive care unit.^8^ One question that still needs to be raised is that the original paper about the score system by Knaus et al. was published in 1985. Our technical and medical knowledge has improved since then, although the mortality rate may not have decreased. For instance, sepsis has presented the same mortality over the last 20 years.^9^ However, it is hard to believe that the prognoses for all other diseases have remained unchanged over this period. What could explain the finding that after 17 years the mortality among intensive care unit patients remains the same, when we should have been able to make it decrease? Maybe the answer is that patients are older, immunosuppressed or oncological in nature. But all these parameters are contained within the APACHE scoring system. What is debarring the effects of our progress from intensive care units?

Finally, physicians working in intensive care units need to note that most of the progress in how to treat diseases is coming from reductions in iatrogenic complications. These have been very well correlated with mortality. There have been reductions in catheter complications, due to catheterization only being indicated when really necessary. It is very easy to avoid or reduce the incidence of nosocomial infections, and this is not expensive to do.^10-12^ Also, ventilation has been improved through reductions in barotrauma and volutrauma.^12,13^ Thus, most of our important evolution in improving patient prognosis is very simple and not expensive. Even quality improvement programs produce a better outcome and reduce costs in a tertiary care unit.^14^
